# Functional characterization of a novel transcript of ERCC1 in chemotherapy resistance of ovarian cancer

**DOI:** 10.18632/oncotarget.20482

**Published:** 2017-08-24

**Authors:** Jia Liu, Lin Zhang, Ping Mao, Guoqiang Jiang, Likun Liu, Jing Wang, Wei Yang, Lawrence Owusu, Weiling Li

**Affiliations:** ^1^ Department of Biotechnology, Dalian Medical University, Dalian, Liaoning, 116044, China; ^2^ Academy of Integrative Medicine, Dalian Medical University, Dalian, Liaoning, 116044, China; ^3^ Department of General Surgery, The People’s Hospital of Liaoning Province, Shenyang, Liaoning, 110016, China; ^4^ Department of Pharmacy, The First Affiliated Hospital of Zhengzhou University, Zhengzhou, Henan Sheng 450000, China; ^5^ Department of Pharmaceutical Sciences, College of Pharmacy, University of South Florida, Tampa, FL 33612, USA

**Keywords:** larger ERCC1 transcript, cisplatin resistance, ovarian cancer, MAPK pathway

## Abstract

Approximately 15-20% of ovarian cancer patients receiving platinum-based chemotherapy are primary platinum-resistant. Identification of these patients and transfer to other more effective therapy could reduce the morbidity of ovarian cancer. ERCC1 is a DNA repair gene which can complex with XPF to repair cisplatin-induced DNA damage and cause chemotherapy resistance. In this study, we found a novel ERCC1 transcript initiated upstream of the normal transcription initiation site. The expression of this larger ERCC1 transcript dramatically increased following cisplatin treatment in ovarian cancer cells and was regulated by the MAPK pathway. This phenomenon conferred enhanced cisplatin resistance on ovarian cancer cells, and was confirmed with chemosensitive and chemoresistant patients’ samples. Our data suggested that larger ERCC1 transcript levels correlated with the outcome of platinum-based chemotherapy.

## INTRODUCTION

Epithelial ovarian cancer (EOC) is the leading cause of death from gynecologic malignancies in the world [1]. The first line therapy for ovarian cancer patients is usually traditional surgery followed by combination platinum-based chemotherapy which may result in complete clinical remission in up to 75% of cases [2]. Most of platium-sensitive patients will eventually become refractory to the treatment. All recurrent ovarian cancer patients develop platinum-resistance over time in terms of secondary platinum-resistance [3]. The remaining 15-20% of patients will however not respond to the initial chemotherapy [4]. Currently, there is no reliable diagnostic biomarker for the resistant patients [5]. Thus, it remains urgent to investigate the underlying mechanisms of chemotherapy resistance and to identify the patients who are resistant to traditional platinum-based chemotherapy to redirect them to alternative therapy.

Nucleotide excision repair (NER) mechanisms are used in the repair of DNA lesions including those produced by chemotherapeutic agents [6]. NER has been strongly linked to cisplatin resistance [7]. Excision repair cross complementation group-1 (ERCC1) is one of the critical genes in NER pathway [8]. The gene for ERCC1 is located on chromosome 19q13.2-q13.3 and has 10 exons spread over 15kb of the genome [9]. It forms a hetero-dimer with XPF to incise the 5’ side of the DNA lesion [10, 11]. A number of studies have correlated ERCC1 levels with DNA repair capacity [12–14]. Higher expression level of ERCC1 reduced the sensitivity of ovarian cancer [15] and other cancers [16] to cisplatin. However, the clinical relevance of ERCC1 as potential predictor for platinum-resistance has been very controversy been discussed in the past in general, especially with regard to immunohistochemical ERCC1 protein detection [17].

Epigenetic changes and alternative splicing of specific genes allow cancer cells to become resistant to chemotherapeutic drugs [18]. ERCC1 gene has several types of transcript variants [19]. A 42-bp spliced sequence in the 5’-UTR of ERCC1 gene has been shown to influence the response of ovarian cancer to cisplatin therapy [20]. ERCC1 exon VIII alternative splicing can also regulate cisplatin-resistance in ovarian cancer [21]. These data suggest that ERCC1 transcript variants may influence the sensitivity of ovarian cancer to chemotherapy.

The MAPK pathway has an important role in regulation of cell differentiation and growth [22]. It has been reported that ERK, one of the kinases in the MAPK pathway, was activated in response to a series of DNA-damaging agents, including cisplatin [23]. Previous studies also indicated that MAPK pathway regulated ERCC1 expression by epidermal growth factor [24]. In addition, cisplatin-induced ERCC1 expressions decreased by blocking ERK activation in lung cancer cell lines [25]. Therefore, we suspected that cisplatin could induce Larger ERCC1 expression by the MAPK pathway.

In this study, we identified a novel ERCC1 transcript in ovarian cancer cells, originating from upstream of normal ERCC1 transcriptional start site and investigate its role in platinum-based chemotherapy.

## RESULTS

### A novel larger ERCC1 transcript variant in ovarian cancer cells

**Figure 1:**
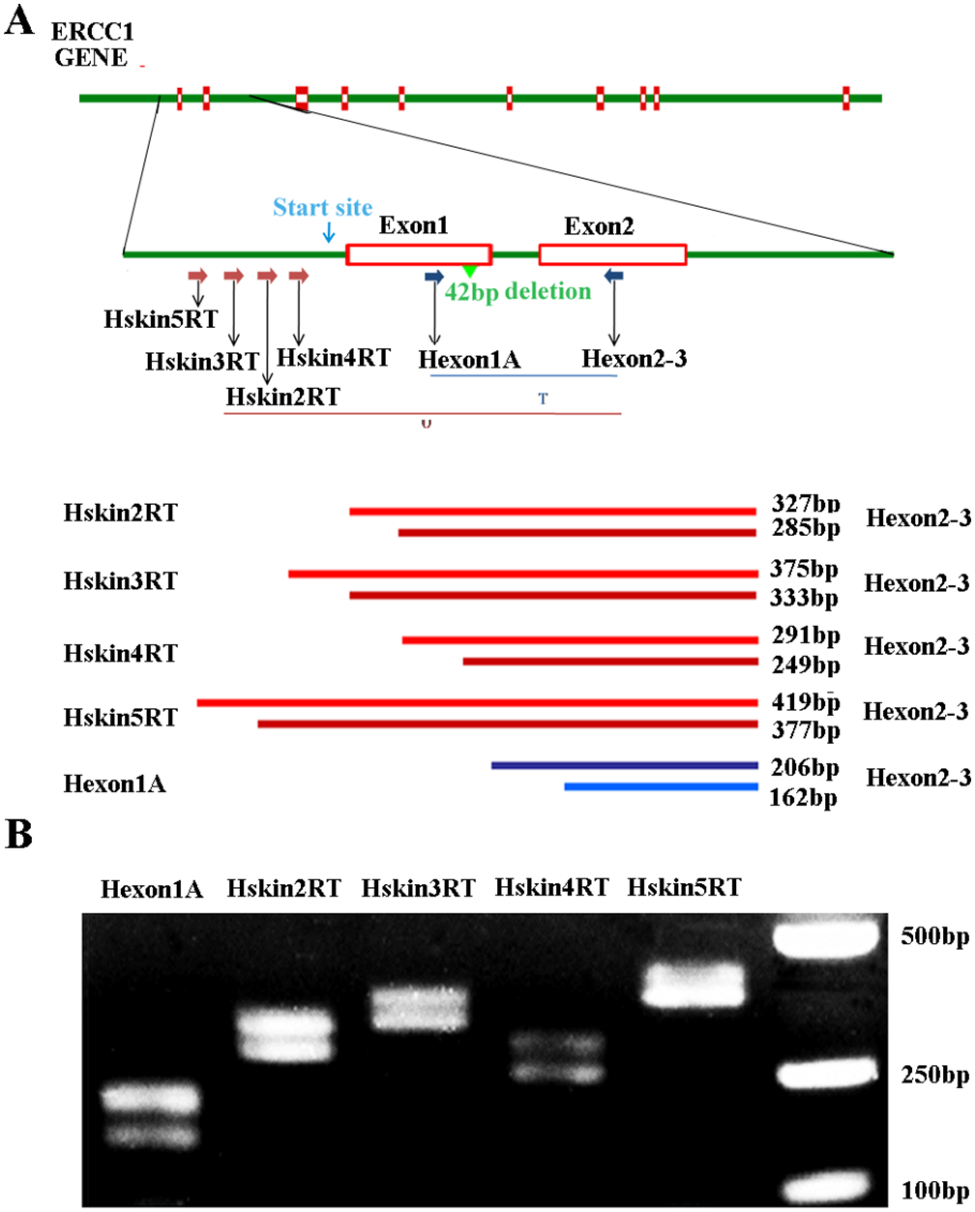
An upstream ERCC1 transcript in human cell lines **(A)** Schematic of human ERCC1 gene showing the exons, location of the normal transcription initiation site (green arrow), transcriptional start site (blue arrow) and presumed location of the upstream initiation site (red arrow). The positions of primers used to detect all ERCC1 transcripts (T) and predicted sizes of PCR products are indicated. The positions of the various primers used to detect the upstream ERCC1 transcripts (U) and predicted size of PCR products are also indicated. **(B)** RT-PCR of mRNA extracted from human ovarian cancer A2780 cells. All ERCC1 transcripts were detected with primer pair of Hexon1A and H-exon 2-3. ERCC1 transcripts initiating upstream were detected with primer pairs of hskin2RT, hskin3RT, hskin4RT, hskin5RT and H-exon2-3.

Previous study showed that a larger ERCC1 transcript encoded ERCC1 protein in mice and this novel transcript originated from an upstream promotor [26]. No such transcript has however been clearly demonstrated in human cells. We, therefore investigated the larger ERCC1 transcript in human ovarian cancer cell A2780 by RT-PCR. Several novel forward primers located upstream of the normal human ERCC1 initiation site were designed as shown in Figure 1A. If novel ERCC1 transcripts originating upstream existed, these primers in combination with reverse primer H-exon 2-3, which is located in ERCC1 exon 2, should generate PCR products from 291bp to 419bp. Our data showed that two bands, 42bp apart, were detected with all primer pairs. Two separated bands were generated by alternative splicing of a 42bp region in ERCC1 exon1 which has been reported previously [27]. The sizes of the gel bands with the different primer pairs agreed with our prediction (Figure 1B). This indicated that the novel larger ERCC1 transcript originating upstream of ERCC1 existed in human ovarian cancer cells. We have also observed the presence of the larger ERCC1 transcript in other human cells such as melanoma, keratinocyte and fibroblast cells (Data not shown). This novel ERCC1 transcript may therefore not be specific to ovarian cancer cells.

**Figure 2:**
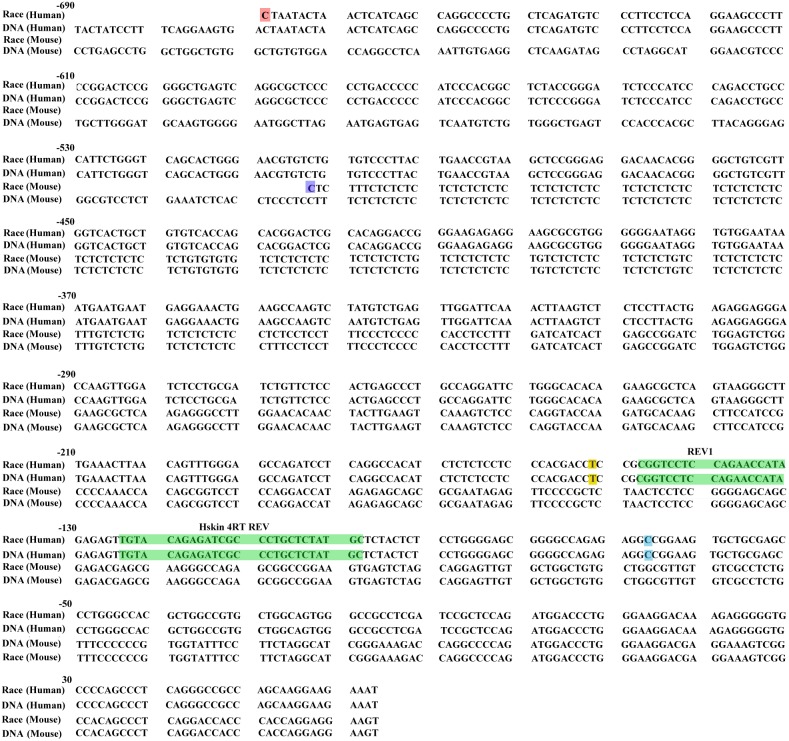
Start points of human ERCC1 larger transcript by 5′ RACE The sequence of the longest 5′ RACE clone determined using human ovarian cancer cell line A2780 is shown, aligned against the mouse larger ERCC1 5′ RACE sequence, human and mouse genomic DNA sequence. The sequence is numbered from the normal translation start site in light blue, with intron 1 and 42bp spliced sequence omitted. The start points of the human larger ERCC1 5′ RACE clones are indicated in red. The start points of the mouse larger ERCC1 5′ RACE clones are indicated in purple. The Hskin 4RT REV and REV1 primers used in our 5′ RACE assay are indicated in green. TSS, normal human transcription start site is shown in yellow at -152bp.

To map the 5’ end of this larger ERCC1 variant, we performed 5’ RACE assay on the ovarian cancer cells using the ERCC1 specific reverse primers from upstream of the normal transcriptional start site. The 5’ RACE sequencing results showed that ERCC1 5′ RACE endpoints determined on the mRNA of human ovarian cells were much further upstream than the normal transcriptional start site. The sequences of 20 independent 5′ RACE clones are shown in Figure 2 aligned alongside mouse larger ERCC1 5′ RACE sequence [28], the human and mouse genomic DNA sequence. The endpoint of the longest clone mapped to -602bp upstream of the normal transcriptional start site (Figure 2).

### Cisplatin induces the expression of larger ERCC1 in ovarian cancer cells

**Figure 3:**
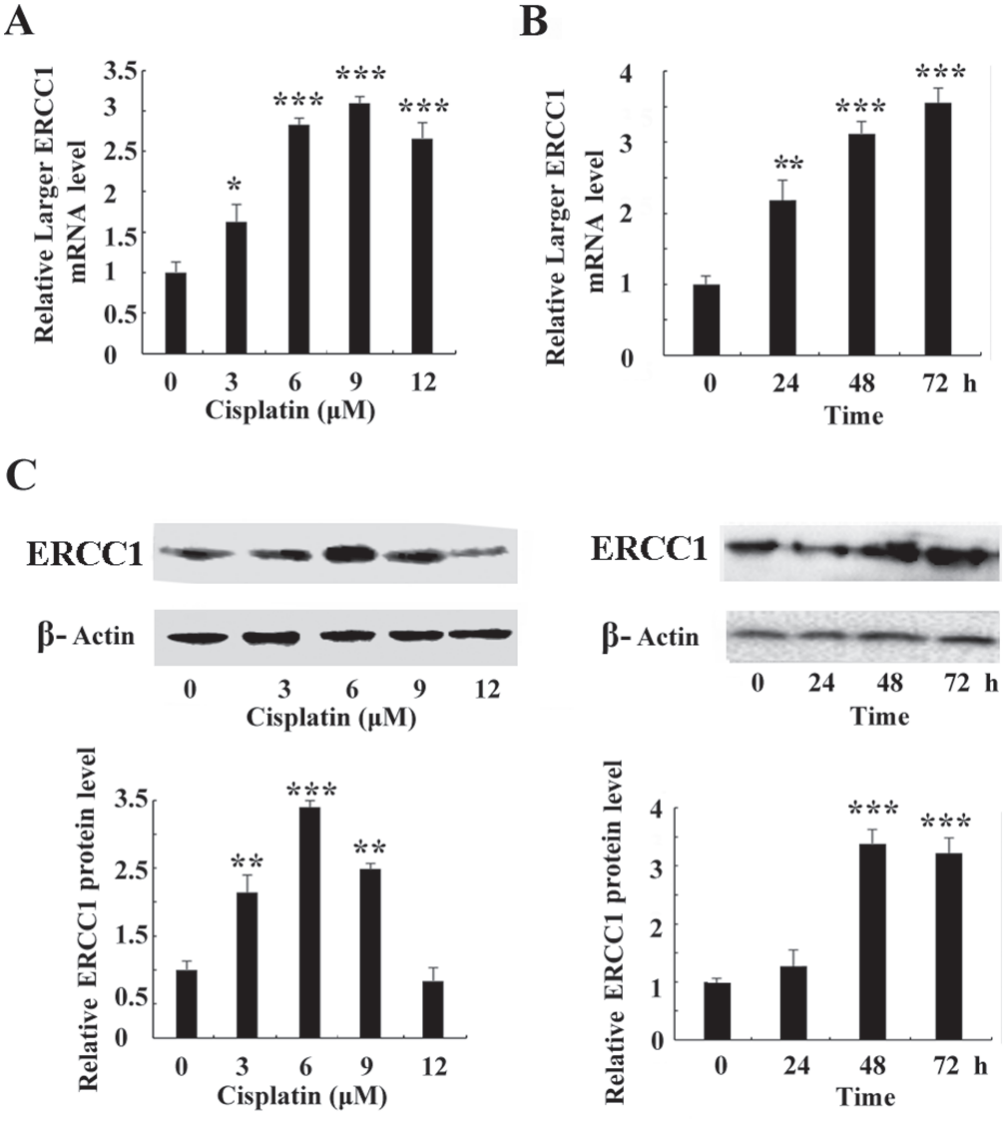
Cisplatin induces the expression of larger ERCC1 in ovarian cancer cells **(A)** A2780 cells were treated with cisplatin (0, 3, 6, 9 or 12 μM) for 24 h. Quantitative real-time PCR assay showed mRNA level of larger ERCC1 after cisplatin treatment. **(B)** A2780 cells were treated with cisplatin (6 μM) for 24 h, 48 h or 72 h. Quantitative real-time PCR assay showed mRNA level of larger ERCC1 following cisplatin treatment at different time point. **(C)** A2780 cells were treated with cisplatin for 48h. Western blot showed protein level of ERCC1 after cisplatin treatment. A2780 cells were treated with cisplatin (6 μM) for 24 h, 48 h or 72 h. Western blot showed protein level of ERCC1 following cisplatin treatment at different time point. Three independent experiments were performed. Student’s t-test, * p<0.05, ** p<0.01, *** p<0.001.

The expression of larger ERCC1 following cisplatin treatment was examined by quantitative real-time PCR (Figure 3). Larger ERCC1 expression was induced by cisplatin (0, 3, 6, 9 or 12 μM) and the peak was 2.8-fold compared to the control group at 6 μM. Our data also indicated that the expression of larger ERCC1 time-dependently increased following cisplatin treatment (6 μM) (Figure 3B). The peak was 3.5-fold compared to the control group at 72 h. Although the LERCC1 mRNA expression peak of the survival cells was at 72h, most of cells were dead because of the hyper toxicity of cisplatin. Therefore, we determined to treat cells with cisplatin (6 μM) for 48 h in the following experiments. Similar to the transcriptional data, our data indicated that cisplatin treatment induced ERCC1 protein expression in ovarian cancer cells (Figure 3C). All together, cisplatin treatment induced a time- and dose- dependent larger ERCC1 increase in ovarian cancer cells. Larger ERCC1 expression may be related to cisplatin resistance in ovarian cancer cells.

**Figure 4:**
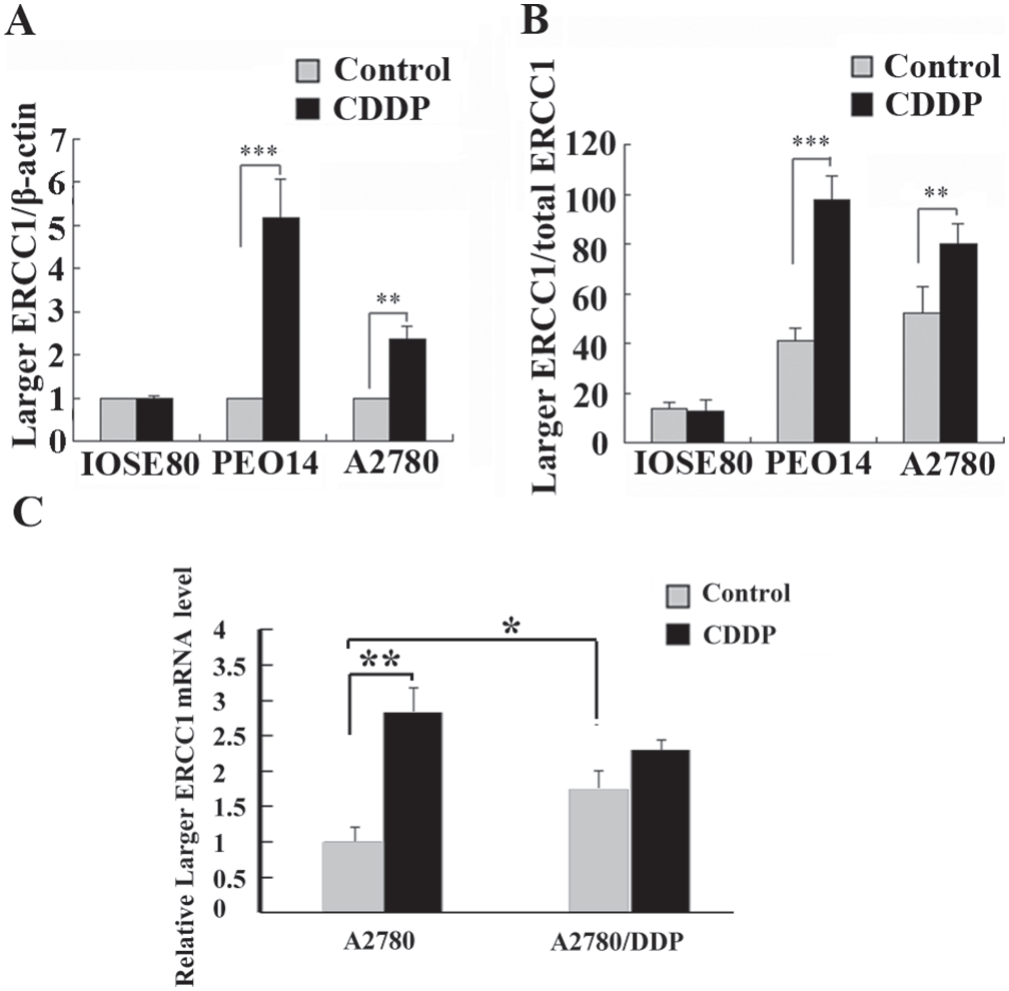
Comparison of the larger ERCC1 expression by cisplatin in different ovarian cells **(A)** Ovarian normal cells ISOE80, ovarian cancer cells PEO14 and A2780 were untreated, or treated with cisplatin (6 μM) for 48 h. Histogram shows the mean level (± SD) of larger ERCC1 mRNA relative to β-Actin determined from three independent experiments by qRT-PCR. Data were normalized to the mean level of larger ERCC1 relative to β-Actin in the controls. **(B)** Histogram shows the ratio (± SD) of larger ERCC1 mRNA relative to total ERCC1 mRNA determined from three independent experiments by qRT-PCR. Larger ERCC1 mRNA and total ERCC1 mRNA levels were each calculated relative to β-Actin. **(C)** Platinum-sensitive ovarian cancer cells A2780 and platinum-resistant A2780/DDP were untreated, or treated with cisplatin (6 μM) for 48 h. Histogram shows the mean level (+ SD) of larger ERCC1 mRNA relative to β-Actin determined from three independent experiments by qRT-PCR. Data were normalized to the mean level of larger ERCC1 relative to β-Actin in the A2780 control. Student’s t-test, * p<0.05, ** p<0.01, *** p<0.001.

To further investigate the relationship between the larger ERCC1 transcript and cisplatin resistance, the expressions of the larger ERCC1 transcript following cisplatin treatment in platinum-sensitive ovarian cancer cells PEO14 and A2780 and normal ovarian epithelial cells ISOE80 were examined. Cisplatin treatment induced approximately 5.3-fold and 2.6-fold increase in larger ERCC1 mRNA expression in ovarian cancer cells PEO14 and A2780 respectively (Figure 4A). However, the expression of the larger ERCC1 transcript in normal epithelial ovarian cells IOSE80 was not significantly affected by cisplatin (Figure 4A). In addition, the ratios of larger ERCC1 to total ERCC1 mRNA following cisplatin treatment in ovarian cancer cells PEO14 and A2780 indicated increases from 41% to 98% and 54% to 79% respectively (Figure 4B). However, the relative abundance of larger ERCC1 in normal ovarian epithelial cells ISOE80 decreased slightly following cisplatin treatment (Figure 4B). It suggested that cisplatin selectively induced the expression of the larger ERCC1 transcript in ovarian cancer cells.

Moreover, we compared the expression of larger ERCC1-transcript between platinum-sensitive A2780 cells and platinum-resistant A2780/DDP cells (Figure 4C). The expression of larger ERCC1 was much higher in A2780/DDP cells than in A2780 sensitive cells. It was suggested that larger ERCC1 transcript was possible to be stably up-regulated in platimum-resistant lines. Our data also indicated that cisplatin induced the larger ERCC1 transcript in platinum-sensitive A2780 cells but not significantly affect that in platinum-resistant A2780 cells. We considered that the resistant cells might originally express high level of larger ERCC1 transcript and leave little increase space.

### Overexpression of larger ERCC1 transcripts enhance cisplatin resistance

**Figure 5:**
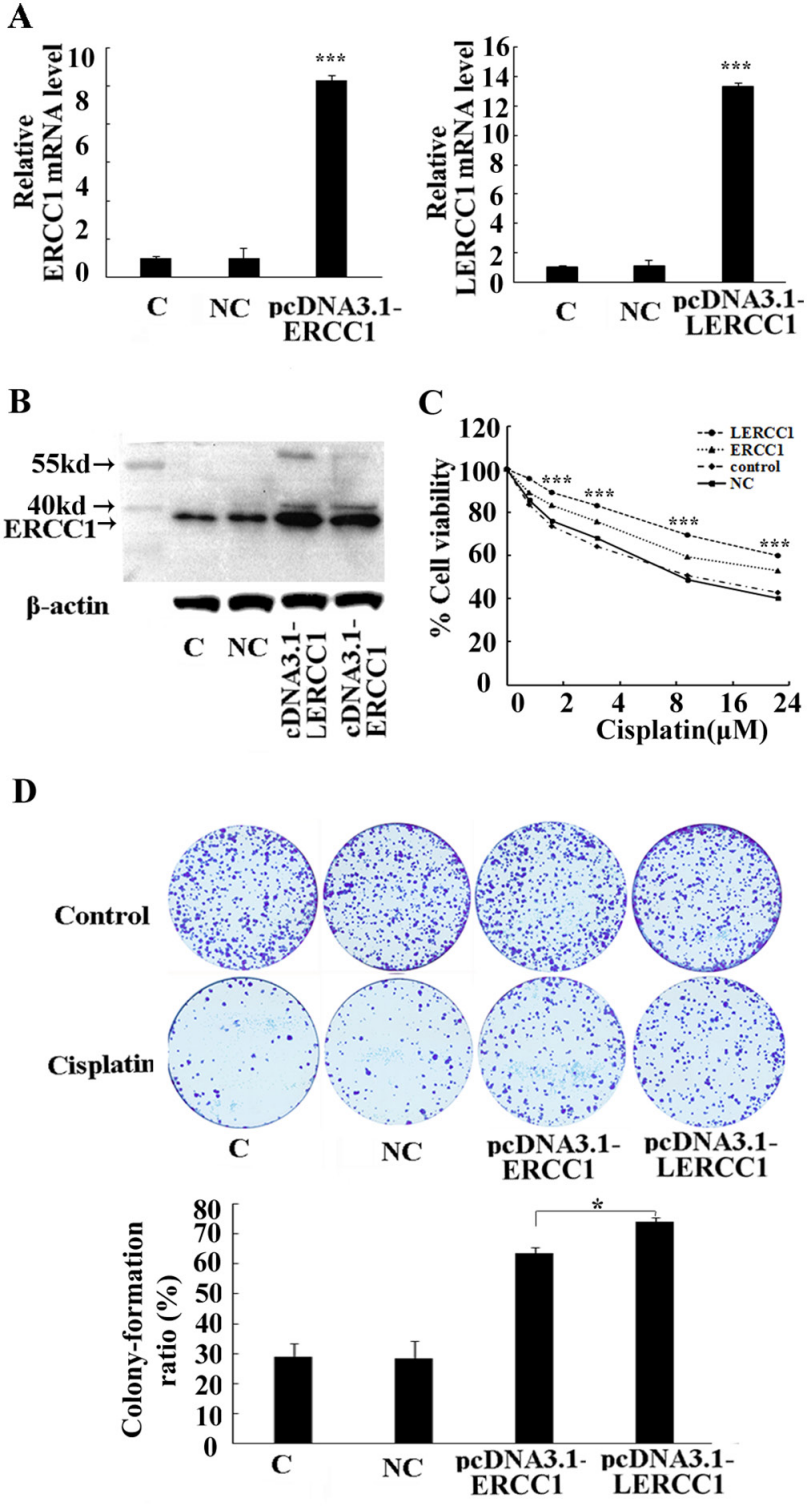
Overexpression of the larger ERCC1 transcript induced the resistance to cisplatin A2780 cells were transfected with either pcDNA3.1-LERCC1, pcDNA3.1-ERCC1 or pcDNA3.1 vector as the negative control (NC). **(A)** The expression of the larger or total ERCC1 mRNA was detected by qRT-PCR. Histogram showed mean levels (+ SD) of the larger or total ERCC1 mRNA against β-Actin. **(B)** The expression of ERCC1 protein was detected by Western blotting. Histogram showed the levels of ERCC1 relative to β-Actin. **(C)** The cells were treated with cisplatin (6 μM) for 48 h. The cells' viability was detected by MTT assay and expressed relative to that of non-transfected cells. **(D)** The cells were treated with cisplatin (2 μM) for 15 days. The surviving colonies were stained and counted. The colony numbers were counted and the colony formation ratio was calculated according to the formula: Colony formation ratio (%) = (number of colonies/number of cells seeded)×100. Three independent experiments were performed.* P<0.05, ** P<0.01, *** P<0.001, Student’s t-test.

To further investigate the roles of larger ERCC1 on chemotherapy resistance in ovarian cancer, A2780 cells were transfected with plasmids containing the larger ERCC1 transcript, wild-type ERCC1 or empty plasmid as the negative control. The larger or total ERCC1 mRNA expression in transfected cells significantly increased compared to the negative control (Figure 5A). The ERCC1 protein level was also elevated by either the larger or wild-type ERCC1 transfection (Figure 5B). MTT and colony formation assay demonstrated that overexpression of the larger or wild-type ERCC1 similarly increased resistance to cisplatin-mediated growth inhibition. However, pcDNA3.1-Larger ERCC1 transfected cells were more resistant to cisplatin treatment than pcDNA3.1-ERCC1 transfected cells (Figure 5C and 5D). Based our data, the novel larger ERCC1 transcript maybe the main ERCC1 transcript involved in the regulation of ovarian cancer cells resistance to chemotherapy.

### Overexpression of larger ERCC1 transcripts decreased DNA damage caused by cisplatin

**Figure 6:**
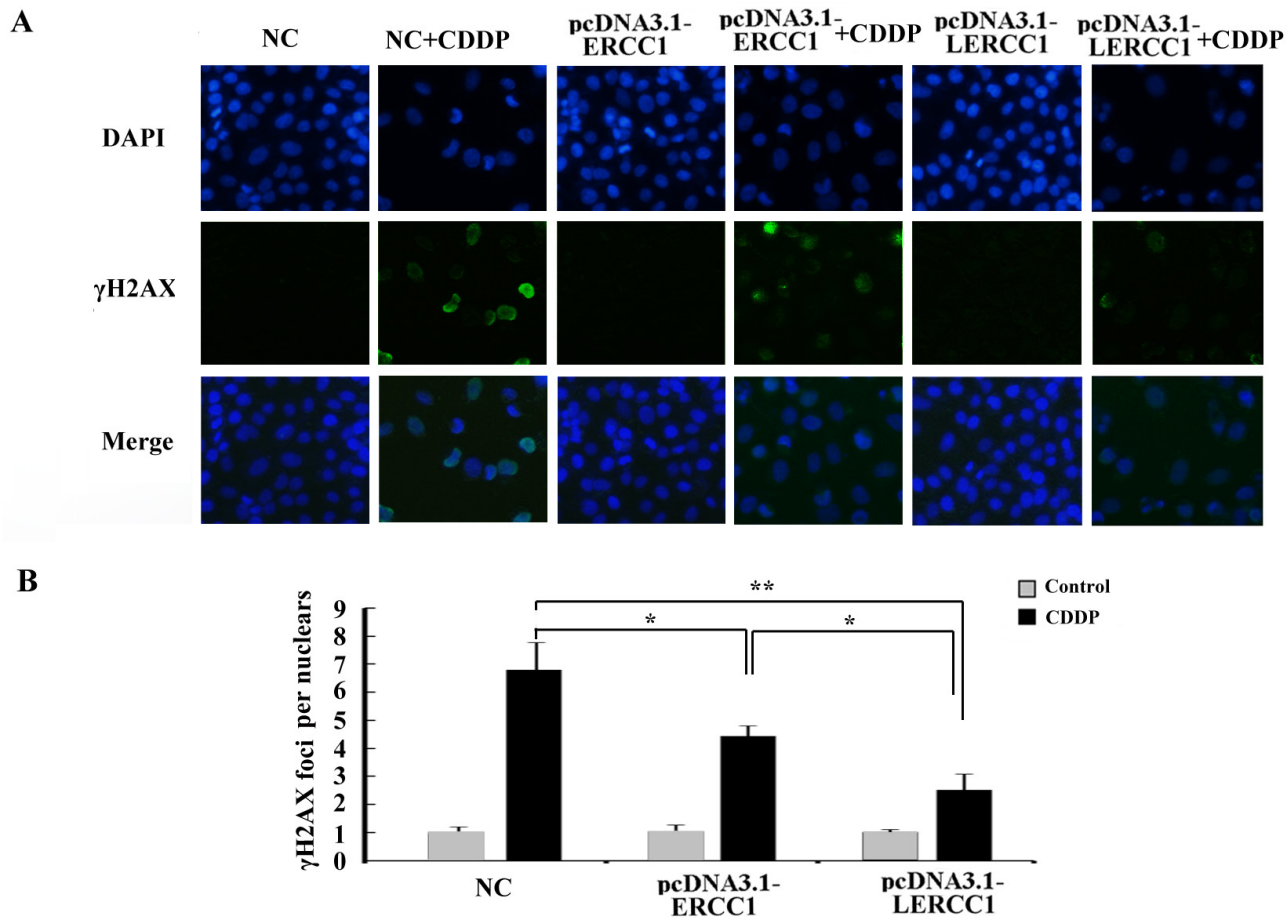
Overexpression of larger ERCC1 transcripts decreased DNA damage caused by cisplatin **(A)** DNA damage in A2780 cells was examined by Immunofluorescence staining. The cells were transfected with pcDNA3.1-LERCC1, pcDNA3.1-ERCC1 or pcDNA3.1 vector as the negative control (NC), and then examined for γ-H2AX foci formation and expression following cisplatin (6 μM, 48 h) treatment. **(B)** IF analysis of γH2AX foci in nuclei of cells (n = 30) by Image J software in three independent assays. Student’s t-test, * p<0.05, ** p<0.01, *** p<0.001.

Larger ERCC1 could have a stronger influence on platinum-resistance. To further detect the DNA-repair capacity of the larger ERCC1 compared to the conventional ERCC1, we detected the expression of phosphorylated H2AX (γ-H2AX) which was an early response to DNA damage and considered as a marker of double strand break. Compared to the conventional ERCC1, overexpression of Larger ERCC1 could more effectively reduced cisplatin induced DNA damage (Figure 6).

### Expression of larger ERCC1 is regulated by the MAPK pathway

**Figure 7:**
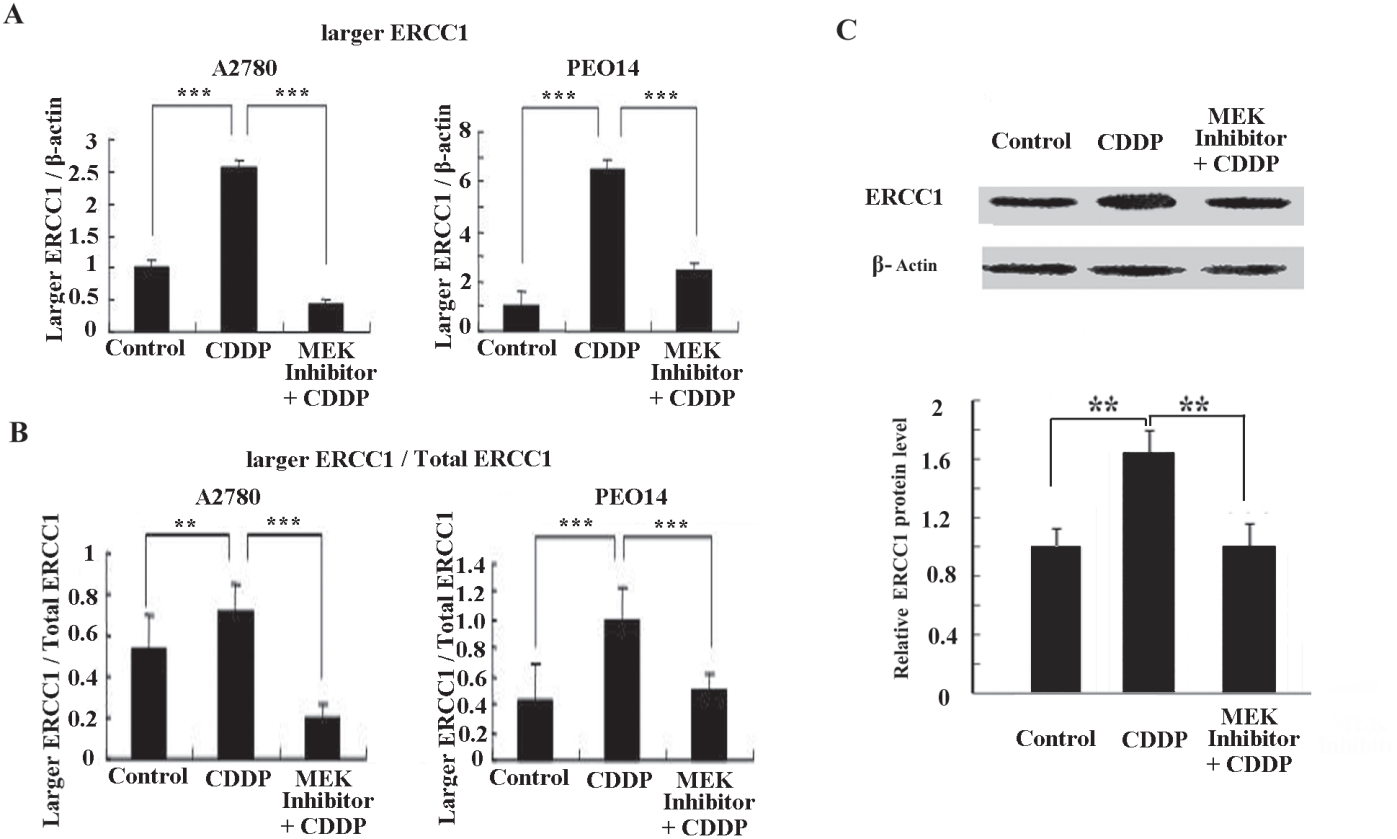
Expression of the larger ERCC1 transcript is regulated by the MAPK pathway Ovarian cancer cells A2780 and PEO14 were either untreated or treated with cisplatin (6 μM), or treated with both cisplatin and MEK inhibitor PD0325901 (1 μM) for 48h. The MEK inhibitor was added 30 min before cisplatin treatment. **(A)** Histogram shows the mean level (+ SD) of larger ERCC1 relative to β-Actin determined from three independent experiments by qRT-PCR. Data were normalized to the mean level of larger ERCC1 relative to β-Actin in the controls. **(B)** Histogram shows the mean ratio (+ SD) of larger ERCC1 mRNA over total ERCC1 mRNA determined from three independent experiments by qRT-PCR. Larger ERCC1 mRNA and total ERCC1 mRNA levels were each calculated relative to β-Actin. **(C)** Western blot showing protein levels of ERCC1 in A2780 cells treated with cisplatin (6 μM) or both cisplatin and PD0325901 (1 μM) for 48h. Histogram showing mean level of ERCC1 expression from three independent experiments after treatment. ** P < 0.01; *** P < 0.001, Student’s t-test.

Many studies have demonstrated an activation of the MAPK pathway in response to cisplatin treatment [29, 30]. Our previous study also indicated that ERCC1 induction by cisplatin was dependent on the MAPK pathway in melanoma cells [31]. Therefore, we probed whether the MAPK pathway activation was responsible for induction of the larger ERCC1 transcript in ovarian cancer cells following cisplatin treatment. A specific inhibitor of MEK1/2, PD0325901, which is highly selective in preventing ERK activation, was used to evaluate the relationship between the MAPK pathway and the larger ERCC1 induction (Figure 7A). We noticed that the larger ERCC1 transcription expression or the ratios of larger ERCC1 relative to total ERCC1 mRNA following cisplatin treatment was efficiently inhibited by the MEK inhibitor (Figure 7B). Our western-blotting data also showed that blocking the MAPK pathway lead to reduction of ERCC1 protein induced by cisplatin (Figure 7C). Thus ERK activation may play an important role in the regulation of larger ERCC1 induction by cisplatin in ovarian cancer cells.

### Expression of larger ERCC1 transcript correlates with platinum chemotherapy responses in ovarian cancer patients

**Figure 8:**
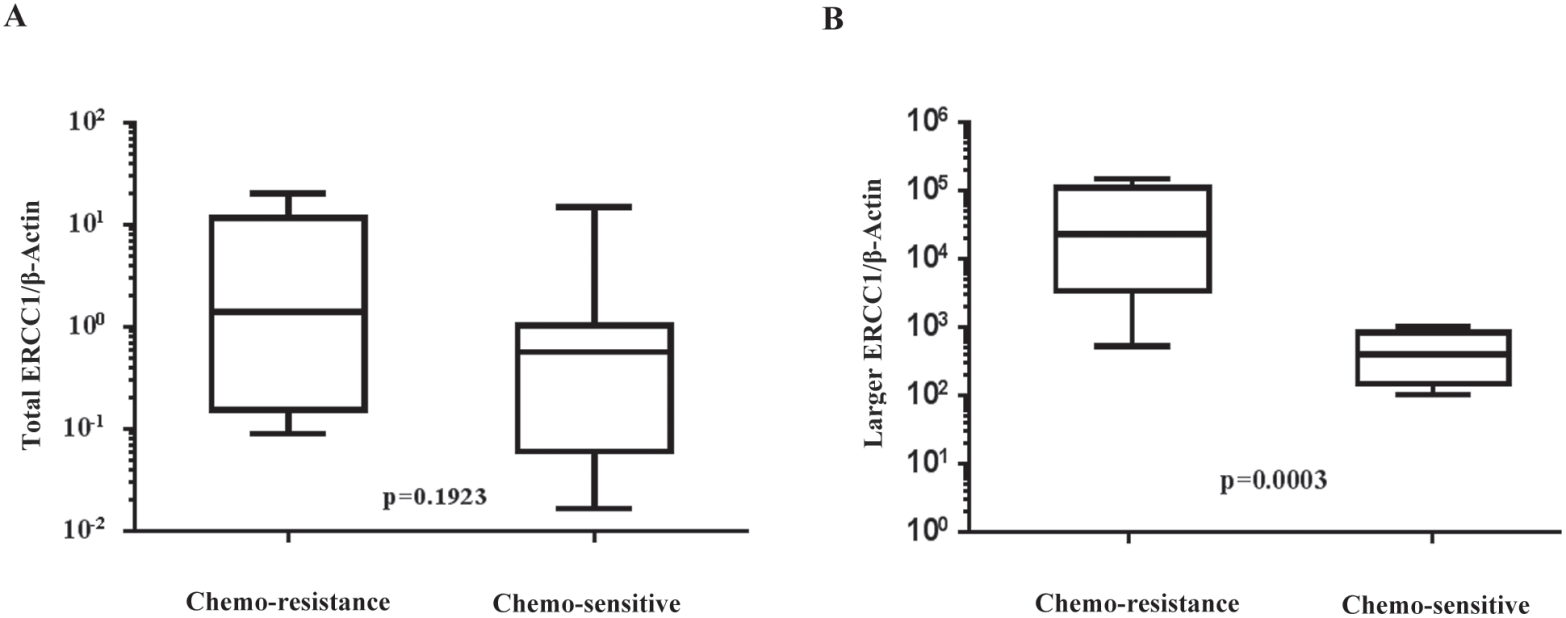
Comparison of normal and larger ERCC1 transcript expression in chemoresistant and chemosensitive patients’ specimen Expression of normal and larger ERCC1 transcript from twenty-three ovarian cancer patients were detected by qRT-PCR. Seven were from chemoresistant patients and sixteen were from chemosenstive patients. **(A)** Expression of total ERCC1 mRNA relative to β-actin inchemoresistant group and chemosenstive group, P=0.1923, Mann-Whitney test. **(B)** Expression of larger ERCC1 mRNA relative to β-actin in chemoresistant group and chemosenstive group, P=0.0003, Mann-Whitney test.

The clinical and pathological characteristics of the subjects in our cohort are shown in Table 1. Of the 23 patients, 16 were chemosensitive and 7 were chemoresistant. The larger ERCC1 transcript and total ERCC1 mRNA expression levels (normalized to β-actin as internal control) in ovarian cancer tissues of these patients were evaluated by real-time PCR. Our data showed that the total ERCC1 mRNA expression levels were similar in chemosensitive and chemoresistant patients (P=0.1923, Figure 8A). However, the expression of larger ERCC1 was significantly higher in the chemoresistant group compared to chemosensitive group (P=0.0003, Figure 8B).

**Table 1:** Clinical characteristics

Characteristic n=23	Drug sensitivepatients (n=16)	Drug resistantpatients(n=7)	p Value
Age (years)			0.819
Median	57.5	52	
Range	48-73	45-68	
FIGOstage			0.45
I-II	3 (19)	0 (0)	
III-IV	13 (81)	7 (100)	
Grade			0.514
1	6 (38)	3 (43)	
2	0 (0)	1 (14)	
3	4 (24)	0 (0)	
ND	6 (38)	3 (43)	
Histologic subtype			0.358
Serous adenocarcinoma	15 (94)	6 (86)	
Mucinous adenocarcinoma	0 (0)	0 (0)	
Endometrioid adenocarcinoma	0 (0)	0 (0)	
Mixed serous mucinous cystadenocarcinoma	1 (6)	1 (14)	
CA125 at diagnosis(U/ml)			<0.001
Median	821	1404	
Range	26-2169	1032-4648	

Data was analyzed by Chi-square test.

FIGO international federation of gynecology and obstetrics, ND not determined.

## DISCUSSION

Cisplatin is one of the most widely used chemotherapeutic agents for the treatment of ovarian cancer [32]. However, the efficacy of cisplatin is hampered by resistance of ovarian cancer cells to its cytotoxicity [33]. ERCC1 plays an important role in recognizing and removing cisplatin- induced DNA adducts and so repairs DNA lesions [34]. Previous study has identified a novel 1.5kb mouse ERCC1 transcript [28]. This novel transcript originated from about 400bp upstream of the normal Ercc1 promoter [30]. However, whether similar novel transcripts existed in human ovarian cells, and the potential functions are yet to be clarified.

In the present study, we have showed that the larger ERCC1 transcript also existed in human ovarian cell lines. The ERCC1 5′RACE endpoints determined on the RNA of human ovarian cell lines were mapped to around 600bp upstream of the normal transcriptional start site. This novel transcript is similar to the novel ERCC1 transcript in mouse skin which mapped to 400bp upstream of the normal transcriptional start point [30]. Whether there is another promoter that regulates the expression of this novel larger ERCC1 needs further investigation.

ERCC1 levels influence DNA repair capacity and associate with cellular resistance to cisplatin [35, 36]. The observation that the ratios of larger ERCC1 relative to total ERCC1 mRNA significantly increased in ovarian cancer cells but not normal cells following cisplatin treatment implied cisplatin may preferentially induce the expression of larger ERCC1 rather than the regular transcript in ovarian cancer cells. In addition, we found platinum-resistant cells stably over-expressed the larger ERCC1 transcript compared to sensitive cells. All together, our data suggested that the novel larger ERCC1 transcript may be related to cellular resistance to cisplatin for ovarian cancer patients.

Further investigations on the functions of the larger ERCC1 transcript indicated that overexpression of the larger ERCC1 transcript in ovarian cancer cells could increase cell viability and stimulate colony formation to protect ovarian cancer cells from cisplatin induced DNA damage. The expression of larger ERCC1 transcript has stronger influence on drug sensitivity and behavior of ovarian cancer cells in response to cisplatin (Figure 5C and 5D). This could be due to a slightly better gene turnover of larger ERCC1 (enhanced translation and/or reduced degradation) compared to normal ERCC1. Moreover, our data revealed that overexpression of larger ERCC1 could more effectively reduced cisplatin induced DNA damage than the conventional ERCC1 (Figure 6). Based on these data, it suggested that cisplatin selectively induced the larger ERCC1 expression which contributed to trigger drug resistance.

ERK1/2 is one of the important kinases in the MAPK pathway that regulates cell growth and death, and has been shown to be activated in response to DNA damage agents, including UV radiation, ionizing radiation, alkylating agents and cisplatin [37]. Previous studies have shown that ERCC1 induction by cisplatin was dependent on the MAPK pathway [31] and that blocking ERK1/2 activation decreased cisplatin-induced ERCC1 protein levels in lung cancer and melanoma cell lines [38]. Our data was in agreement with these observations (Figure 7) and thus suggests the induction of larger ERCC1 by cisplatin to be, at least, through the MAPK regulatory pathway. However, how this pathway might preferentially enhance larger ERCC1 expression upon cisplatin treatment is yet to be deciphered.

Previous studies investigating the relationship between ERCC1 expression and clinical outcomes of platinum-based chemotherapy in ovarian cancer have yielded conflicting results. Some suggest prognostic value of ERCC1 in ovarian cancer [36, 39], whereas others express limited clinical significance of the gene in primary ovarian cancer patients [40, 41]. In support of the latter view, we have demonstrated that the larger ERCC1 transcript level but not total ERCC1 is possible to correlate with the sensitivity of ovarian cancer patients to platinum-based chemotherapy (Figure 8). However, we admitted that limitation of the small clinical sample size in this study. We will further explore these results with large-sample-size pharmacological study in the future.

In conclusion, our study identified a novel larger ERCC1 transcript originating from upstream of the normal ERCC1 transcriptional start point. This larger ERCC1 transcript was induced by cisplatin treatment through the MAPK pathway. More importantly, the larger ERCC1 transcript enhanced ovarian cancer cells resistance to cisplatin treatment. Thus larger ERCC1 transcript levels may correlate with the outcome of platinum-based chemotherapy.

## MATERIALS AND METHODS

### Cell lines and reagents

Human ovarian cancer cells A2780 and A2780/DDP were obtained from the American Type Culture Collection (ATCC) and PEO14 was kindly provided by Dr. Charlie Gourley (Edinburgh, UK). Both ovarian cancer cell lines were cultured in Dulbecco's modified Eagle's medium (DMEM) complete medium, supplemented with 10% fetal bovine serum (FBS). Normal ovarian epithelial cell line IOSE80 was obtained from Canadian Ovarian Tissue Bank and grown in medium 199 (Invitrogen) and MCDB 105 (Sigma), supplemented with 10% FBS. Rabbit anti-human ERCC1 and β-actin polyclonal antibodies were purchased from Proteintech Group lnc. (Chicago, USA). Cisplatin was purchased from Sigma (St. Louis, MO, USA) and MEK inhibitor PD0325901 was obtained from Selleck (St. Louis, MO, USA). Antibody against γH2AX was purchased from Cell Signaling Technology (MA, USA). Antibody against ERCC1 was purchased from Santa Cruz (CA, USA).

### Tissue collection

Twenty-three surgically resected primary epithelial ovarian cancer samples were collected in accordance with The Code of Ethics of the World Medical Association during the period of 2016 to 2017 at the Affiliated Cancer Hospital of Zhengzhou University. Diagnosis of ovarian cancer was evaluated by pathologists. The Ethical Research Committee of Affiliated Cancer Hospital of Zhengzhou University approved the protocol of this study. All patients formally and voluntarily gave their consent for the study. All patients underwent debulking surgery and subsequent platinum-based chemotherapy. Baseline characteristics and serum CA125 levels were collected. All patients had a follow up ≥ 12 months by serum CA125 and documentation of lesion progression or appearance of new lesions on CT-scan. Chemotherapy resistance or sensitivity was defined as the presence or absence of tumor relapse progression within 6 months, or 6 months after completion of prior platinum-based chemotherapy. Patient characteristics are listed in Table 1. The data were analyzed anonymously.

### RNA isolation

Total RNA was extracted from cells or human tissues using Trizol reagent (Life technologies, California) or Magnetic Total RNA kit (GenePharma, China) according to the manufacturer’s instructions. In briefly, cut 2-5 paraffin sample sections between 5-10 μm. Immediately place the sections into the 1.5 ml microcentrifuge tube. Incubate at 80°C for 15 min to melt the paraffin. Immediately centrifuge at 13,000 × g for 5 min. Add 400 μl RNA Wash Buffer II. Resuspend the Mag-Bind thoroughly by vortexing for 20 sec or pipetting up and down 10-20 times. Place the tube onto the magnet separation device to magnetize the Mag-Bind. Aspirate and discard the cleared supernatant. Remove any liquid drops from tube. Add 73.5 μl DNase I Digestion Buffer and RNase-free DNase I to each sample. Resuspend the Mag-Bind thoroughly by vortexing for 20 sec or pipetting up and down 10-20 times. Incubate at 37°C for 15 min. Add 225 μl GFC Buffer. Mix thoroughly by vortexing for 20 sec or pipetting up and down 10-20 times. Place the tube onto a magnet separation device to magnetize the Mag-Bind. After 3-5 min aspirate and discard the cleared supernatant. Remove the tube from the magnetic separation device. Air dry the Mag-Bind by leaving the tubes on the magnetic separation device for 10 min. Transfer the cleared supernatant containing purified RNA into a new nuclease-free 1.5 ml microcentrifuge tube. Store the purified RNA at -80°C.

### RT-PCR

The RT-PCR was performed in 50 μl reaction containing 0.5 pmol/ul primers, 0.25 mM dNTPs, 2.5 mM MgCl_2_, 100 ng cDNA template, 1U Taq DNA polymerase (Promega), and Taq PCR buffer (Promega). PCRs were carried out by DNA denaturation at 94°C for 5 min, followed by 30 cycles of DNA denaturation at 94°C for 1 min, primer annealing at 50 °C for 1 min and DNA polymerase extension at 72 °C, with a final extension of PCR products at 72°C for 5 min after cycling. All forward and reverse primers used for RT-PCR are mentioned in Table 2.

**Table 2:** All primers used for 5’ RACE assay

Name	Sequence 5'-3'	Description
H-exon 2-3 reverse	ATTTGTGATACCCCTCGACGAG	Normal human ERCC1 transcript reverse primer
Hskin4RT reverse	TGTACAGAGATCGCCCTGCTCTATGC	Larger Human ERCC1 transcript reverse primer
REV1	CGGTCCTCCAGAACCATAGA	Larger Human ERCC1 transcript reverse primer
Oligo dT anchor	GACCACGCGTATCGATGTCGACTTTTTTTTTTTTTTTTV (V=A, C or G)	Anchor primer
PCR anchor	GACCACGCGTATCGATGTCGA	Internal anchor primer

### 5′ RACE assay

Characterization of the 5′ end of the human larger ERCC1 transcript was exerted using 5′/3′ RACE kit (Roche) following the manufacturer’s instruction. In brief, the first strand cDNA was synthesized from total RNA using an ERCC1-specific primer H exon 2-3 with deoxynucleotides and reverse transcriptase. The synthesized cDNA was then purified by High Pure PCR Product kit (Roche) and a homopolymeric A-tail was added to the 3′ end of the cDNA. The tailed cDNA was PCR amplified using a second ERCC1-specific primer Hskin 4RT REV in combination with oligo dT-anchor primer. To further amplify this cDNA, a further round of nested PCR was performed using the internal primers PCR-anchor and REV1. The PCR products were separated by 1.5% agarose gel and cloned into p-GEM T-EASY vector for sequencing analysis. All primers used for 5’ RACE assay are mentioned in Table 2.

### Real time PCR analysis

Real-time quantitative polymerase chain reactions were used to quantify the cDNAs of interest using Taqman^®^ Gene Expression Assay and Applied Biosystems 7900HT Fast Real-Time PCR System. Real-time quantitative polymerase chain reactions were prepared in a 20 μl volume containing 1ul specific 20X Taqman Gene Expression Assay, 10 ul 20X Taqman Gene Expression Master Mix, 4ul cDNA template (10-100 ng), and 5ul RNase-free water. Routine real-time quantitative polymerase chain reaction conditions were: AmpliTaq Gold Enzyme activation at 95°C for 10 min, then 40 cycles of DNA denaturation at 95°C for 10 min, primer annealing and DNA polymerase extension at 60 °C for 60 sec. The primer sequence for the larger ERCC1 transcript was 5'-CTGGGAATTTGGCGACGTAA-3' and 5'-ATGGATGTAGTCTGGGTGCAG-3'.

### Expression vector construction and transfection

cDNAs were reverse-transcribed using TransScript Taq reverse transcriptase and Random primers. The larger and normal ERCC1-coding region were amplified by polymerase chain reaction (PCR). The PCR products were cloned into the constitutive mammalian expression vector pcDNA3.1 containing neomycin selectable marker. A2780 cells were transfected with pcDNA3.1-larger ERCC1, pcDNA3.1-ERCC1 or pcDNA3.1 vector as the negative control using a Lipofectamine 2000 Transfection Reagent (Invitrogen, USA) according to the manufacturer’s protocol. Stable transformants were selected by 500μg/ml G418 (Sigma, USA) treatment. Selection pressure was removed after pools of transfected colonies were isolated.

### Western blot analysis

Cells were harvested and lysed in cold RIPA buffer and quantitated. Proteins were separated by 10% SDS-PAGE and transferred onto nitrocellulose membrane using a semi-dry transfer apparatus for 40min. The membrane was blocked with 5% non-fat milk for 1h and then incubated with the specific primary antibody solution overnight at 4°C. Signal was detected using HRP-conjugated secondary antibodies and visualized by ECL (Thermo Scientific, USA).

### MTT assay

The cells (1×10^4^/well) in 200μl medium containing cisplatin (6 μM) or without cisplatin were seeded into 96-well plate. After 48h treatment, 20 μl MTT (5 mg/mL in PBS) was added to each well and incubated for 4 h. The MTT solution was removed and the formazan crystals were dissolved in 150 μl DMSO. Absorbance of the solution was measured using a Multiskan Ascent plate reader at 540 nm wavelength.

### Colony formation assay

Five hundred cells per 35mm dish were plated before treatment. Then 1ml medium containing or without cisplatin (2 μM) were added into each well. Cells were fixed on day 15 using 70% ethanol, and stained using crystal violet (Sigma, St. Louis, MO, USA) dissolved in 10% ethanol. Number of stable colonies was obtained by counting. Colonies were defined as a minimum of 50 cells in a group

### Immunofluorescence staining

Cells were plated on glass coverslips (Fisher Scientific). After treatment with cisplatin (6 μM), cells were fixed with 4% paraformaldehyde. Following PBS wash, cells were permeabilized using 0.2% Triton X-100, incubated in a blocking solution (PBS, 3% bovine serum albumin), and further incubated overnight at 4°C with primary antibodies anti-γH2AX. Cells were incubated with the appropriate fluorescent-conjugated secondary antibody and DAPI (Sigma Aldrich) was used as a nuclear counterstain. Finally, the coverslips were mounted onto slides with fluorescent mounting medium (Vector Labs) and immediately observed under a confocal microscope (Carl Zeiss, LSM 710). Image J software was used to quantify fluorescent intensity.

### Statistical analysis

Data are presented as the mean ± SD. Student’s t-test or the Mann-Whitney test was performed to estimate statistical differences between groups using GraphPad Prism version 5 (GraphPad Software, Inc, La Jolla, CA, USA). Chi-square test was used to analyze the clinical characteristics by SPSS version 14.0 (SPSS, Chicago, IL, USA). Values of P < 0.05 were considered statistically significant.
